# Health Risk Assessment of Different Heavy Metals Dissolved in Drinking Water

**DOI:** 10.3390/ijerph16101737

**Published:** 2019-05-16

**Authors:** Sajjad Hussain, Muhammad Habib-Ur-Rehman, Tasawar Khanam, Abbas Sheer, Zhang Kebin, Yang Jianjun

**Affiliations:** 1 School of Soil and Water Conservation, Beijing Forestry University, Beijing 100081, China; sajjad.husains786@gmail.com; 2 Institute of Environment and Sustainable Development in Agriculture, Chinese Academy of Agricultural Sciences, Beijing 100081, China; 3 Department of Agronomy, MNS-Agriculture University, Multan 66000, Pakistan; Habib.rahman@mnsuam.edu.pk; 4 Environmental Toxicology Lab, Department of Biosciences, COMSATS University Islamabad, Islamabad 44000, Pakistan; tasawwursatti@gmail.com; 5 Beijing Institute of Technology (BIT), School of Law Beijing, Beijing 100081, China; 3820160061@bit.edu.cn

**Keywords:** trace elements, risk assessment, drinking water, anthropogenic, geologic, mass spectrometry

## Abstract

Water pollution is a major threat to public health worldwide. The health risks of ingesting trace elements in drinking water were assessed in the provinces of Punjab and Khyber Pakhtunkhwa, Pakistan. Eight trace elements were measured in drinking water, using Inductively Coupled Plasma Mass Spectrometry (ICP-MS), and compared with permissible limits established by the World Health Organization (WHO) and the Pakistan Environmental Protection Agency (Pak EPA). In addition, health risk indicators such as the chronic daily intake (CDI) and the health risk index (HRI) were calculated. Our results showed that the concentrations of chromium (Cr), nickel (Ni), and manganese (Mn) were 2593, 1306, and 695 ng/g, respectively, in Lahore and Jhang, while the concentrations of arsenic (As) in Lahore, Vehari, Multan, and Jhang were 51, 50.4, 24, and 22 ng/g, respectively, which were higher than the permissible limits suggested by the WHO. The values of CDI were found to be in the order of Cr > Ni > Mn > Cu > As > Pb > Co > Cd. Similarly, the health risk index (HRI) values exceeded the safe limits (>1) in many cities (eg, Cr and Ni in Lahore and As in Vehari, Jhang, Lahore, and Multan). The aforementioned analysis shows that consumption of trace element-contaminated water poses an emerging health danger to the populations of these localities. Furthermore, inter-metal correlation and principal component analysis (PCA) showed that both anthropogenic and geologic activities were primary sources of drinking water contamination in the investigated areas.

## 1. Introduction

Water is an essential element for life. Freshwater comprises 3% of the total water on Earth. Only a small percentage (0.01%) of this freshwater is available for human use [1]. Groundwater is an important freshwater resource and is in increasing demand for agricultural, industrial, and domestic usage. As per global estimates, although groundwater comprises only 0.61% of the overall water resources of the world, 20% of the freshwater supply is characterized by it [2]. Although groundwater delivers a comparatively pathogen-free source of drinking water, other pollutants leach into it through aquifer rocks and sediments, affecting its chemical quality [3]. Deterioration of groundwater can be due to geogenic and anthropogenic reasons. Precipitation is a principal source of aquifer recharge, and it gathers atmospheric, geogenic, and anthropogenic chemical pollutants as they trickle into groundwater basins through aquifer rocks. Over-pumping has also contributed to shifts in freshwater levels, ultimately affecting groundwater quality as well [4]. Geogenically, these impurities enter groundwater if they are present plentifully in the earth’s crust or in residues surrounding the aquifer, whereas impurities from anthropogenic environments make their way into groundwater by solubilizing in water from rainfall or through irrigation returns that finally enter groundwater [5]. Due to chemical interactions between water and geological environments, numerous chemical compounds are found in groundwater in various concentrations. As a consequence, several research studies have been conducted to assess groundwater quality and the health risks posed by toxic and trace elements such as Cr, Co, Mn, Ni, Zn, Cd, Cu, Pb, and As, mainly from countries such as Pakistan, Bangladesh, India, China, Vietnam, Cambodia, and others in South East Asia [6,7]. Mn, Zn, Cu, and Co are required by the human body for several functions, whereas Cd, Pb, Ni, Cr, and As are poisonous non-essential elements that contribute to numerous human health hazards upon food intake [8]. The toxic effects of these elements include health issues such as abdominal pain, high blood pressure, kidney damage and eventually failure, irritability, skeletal harm and degradation, cancer, nerve damage, headaches, and neurodegeneration and its consequences on the intellectual system. These specific effects, however, depend on the type of contaminant, its concentration, and the cause and span of contact [9]. Although most of these elements are carcinogenic, they adopt diverse pathways and only affect certain organs and systems. Arsenic is believed by the World Health Organization (WHO) to be amongst the worst cancer-causing components present extensively in the environment and has many other adverse health effects including skin lesions, neurological problems, circulatory malfunction, diabetes, hepatic and renal syndromes, respiratory complications, several types of cancer including leukemia, problems with male and female fertility, and mortality as a consequence of chronic diseases [9,10].

Taking into account the health hazards to the population, researchers in Pakistan have taken an interest in the level and extent of contact with such contaminants [11]. Being a developing country and agrarian economy, Pakistan fights with scarcity and quality of water, and relies primarily on groundwater for drinking and irrigation purposes. This is because of easy access to groundwater through wells, hand-pumps, and tube-wells [12]. Therefore, with high concentrations of trace elements, groundwater has the potential to become the main source of exposure or contact to these elements. Unfortunately, the findings of similar studies show that due to the anthropogenic and geogenic environment of Pakistan, water is not safe for intake in various parts of the country with regard to many contaminants, especially As, as per standard concentration parameters given by the World Health Organization (WHO) for drinking water [12,13,14,15]. Although indigenous reports show conflicting data with respect to global standards, only 25.61% of the people in Pakistan have access to safe drinking water [16]. Cr, Ni, Cd, and As are normally found in the earth’s crust and are affiliated with anthropogenic activity as well. Their concentrations are said to have significantly exceeded safe limits, although the results from different areas vary [17]. Pb and Hg are also believed to be emerging from natural and human activity. In Pakistan, studies present outcomes that contradict studies conducted by the PCRWR (Pakistan Council for Research in Water Resources), which show concentrations that are generally within safe limits, while individual studies suggest the existence of groundwater polluted beyond safe limits in different areas. However, studies concerned with these elements in Pakistan are scarce. Mn, Co, Zn, and Cu are all important trace elements that are reported at large to be within the safe limits in Pakistan [14].

Owing to limited financial resources, people in Pakistan are facing a serious problem in the supply of clean and safe drinking water because they directly use underground water for domestic and other applications. In the past, varied and even conflicting results on the safety of groundwater were reported in different population-enriched areas in Pakistan, which highlights the great concern about groundwater quality and the need for further investigation on groundwater contamination and the potential pollution sources. For instance, the Pakistan Council for Research in Water Resources (PCRWR) has declared that Punjab province cities, such as Lahore, Vehari, Multan, Sheikhopura, Bahawalpur, Gujranwala, and Kasur are the most-affected areas with respect to elevated trace metal concentrations [18]. Despite the deplorable conditions in most of the areas, a limited number of researches have been conducted with regard to drinking water contamination and its potential effects on human health, especially in Khyber Pakhtunkhwa (KPK). In addition, more than half of the inhabitants in the Nowshera, Charsadda, Mardan, and Peshawar districts of Khyber Pakhtunkhwa have limited access to clean drinking water [15]. Therefore, the aim of this study was to assess heavy metal contamination in drinking water and the associated health risks in major areas of the Punjab and Khyber Pakhtunkhwa provinces of Pakistan.

## 2. Materials and Methods

### 2.1. Groundwater Sampling and Analysis

#### 2.1.1. Study Area

Pakistan is situated in South Asia, covers 796,095 km^2^, and consists of varying geological features. The geology of Pakistan ranges from high mountains in the north to alluvial plains and even deserts in the south, with five rivers of different origins that irrigate the land and affect sedimentation. The study area is comprised of two provinces: Punjab and Khyber Pakhtunkhwa (KPK). Punjab, the second largest province of Pakistan, mostly consists of fertile alluvial plains of the Indus River. It is a heavily irrigated area, and canals are found throughout the province. In Punjab, most areas experience extremely foggy winters and hot summers, and temperatures range from 2 to 45 °C. KPK sits primarily on the Iranian plateau and has huge Hindu Kush mountains, and its major parts are typically dry. Multiple samples were collected from each site to span the whole area. Between any two sampling points, a minimum distance of 1 km was maintained.

#### 2.1.2. Water Sample Collection and Their Preservation

Representative samples were collected without changing the element concentrations between sampling and analysis. Sterilized, recyclable, disposable conical tubes that had locking screw caps of a 15 mL volume were used to collect samples. Millipore syringe filters with a 0.2 μm pore size were used for the filtration of samples. To eliminate microbial activity, several drops of nitric acid were also added to the samples (decreasing pH to 2). Information regarding sampling date, time, and ID code was given to each sample label. A separate field pro forma was designed in order to record the field observations of each sample and every sampling site. Information that was gathered regarding sampling site contained observations about potential As sources around the sampling range and the prominent health effects of As amongst the locals. A geographical position system (GPS) was used to record the coordinates of sampling sites.

#### 2.1.3. Inductively Coupled Plasma Mass Spectrometry

Agilent 7500 cx ICP-MS (Inductively Coupled Plasma Mass Spectrometry, Agilent Technologies, Santa Clara, CA, USA) was used in determining trace elements concentrations in water samples. Serial dilution of standard stock solution (100 μg/mL, GSB 04-1767-2004), obtained from the NCATN (National Center of Analysis and Testing for Nonferrous Metals and Electronic Materials), was used to prepare calibration solutions. Operating parameters for ICP-MS were set as follows: Carrier gas: 1.1 L/min; radio-frequency (RF) power: 1510 W; nebulizer pump: 0.1 rps; helium gas: 3.5 mL/min; makeup gas: 0.10 L/min.

#### 2.1.4. Quality Control (QC) in the Analytical Procedure

As a quality control, a representative of all samples containing 20 μL of every sample was prepared and injected after every 30 samples. The variation in metal concentrations was <10% for QC samples. In addition to that, standards were calibrated twice, at the start and at the end. Each sample was injected three times by the instrument, and an average was given as a result during every test. All samples were tested thrice, i.e., a pure sample once and fivefold-diluted samples twice, in order to make sure that the results were precise.

#### 2.1.5. Health Risk Assessment

For human health risk assessment, the health risk index (HRI) for all elements in each area was also calculated. Using chronic daily intake (CDI) and the reference dose (RfD) for each element, the HRI was set by the Agency for Toxic Substances and Disease Registry (ATSDR) or the Environmental Protection Agency (EPA). The values for CDI through water ingestion were calculated using a modified equation from the EPA [19].

CDI = C × (DI/BW)(1)

HRI = CDI/RfD(2)

where daily intake (DI) was assumed to be 2 L, body weight (BW) was assumed to be 70 kg, and the mean concentration (C) of elements in samples was taken in μg/L.

#### 2.1.6. Statistical Analysis

Univariate and multivariate statistical analyses such as inter-metal correlation were performed using SPSS (statistical package for the social science, version 22). However, to compare different trace element concentrations in different cities, analysis of variance (ANOVA) was performed using Statistix 8 (version 8.1) and principal component analysis (PCA) was performed in an R environment (R version 3.5.1). The concentration maps of metals in the study area were prepared using the Arc geographic information system (version 10.3.1). Furthermore, the Spearman correlation coefficient matrix for all the selected elements was used.

## 3. Results and Discussion

### 3.1. Drinking Water Contamination

**Figure 1:**
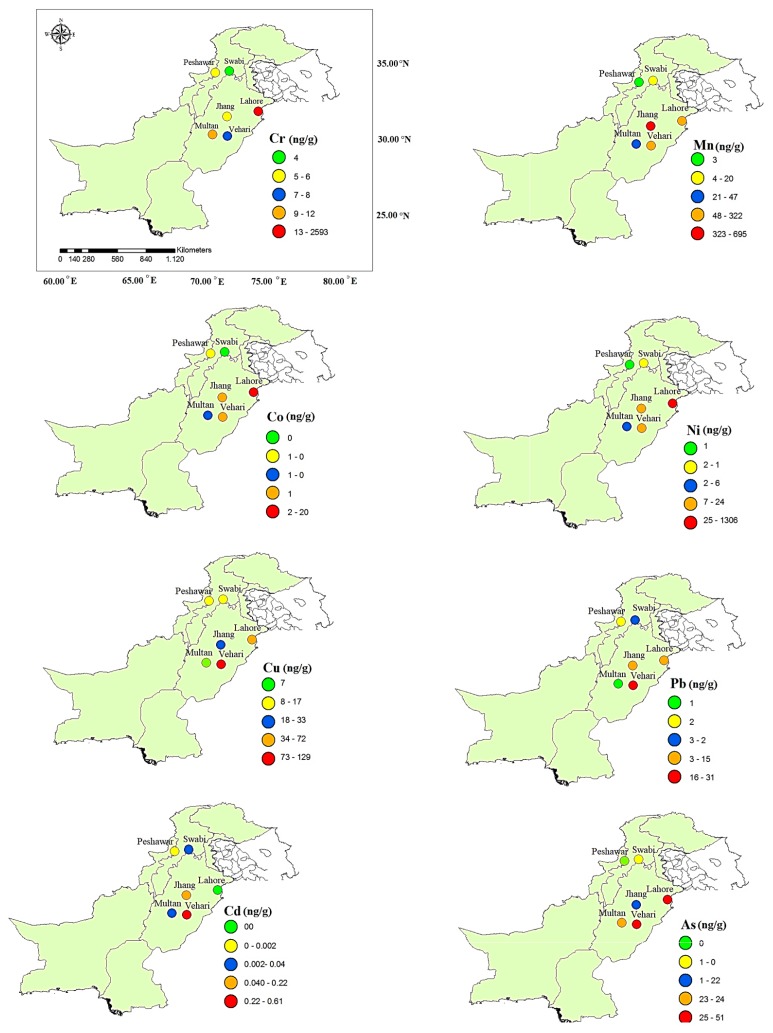
Maps showing mean concentrations of different trace elements in drinking water samples collected from different cities of Pakistan.

In the present study, drinking water quality has been assessed on the basis of the WHO [20] and the Pakistan Environmental Protection Agency (Pak-EPA) [21] standards. The trace elements in some drinking water samples were higher than the WHO guidelines (Figure 1). Trace element concentrations in drinking water samples were found to be in the order of Cr > Ni > Mn > Cu > As > Co > Pb > Cd in Lahore. Moreover, in Jhang and Multan, the patterns found were Mn > Cu > As > Ni > Pb > Cr > Co > Cd and Mn > As > Cr > Cu > Ni > Pb > Co > Cd, respectively. In Vehari and Peshawar, the observed patterns were Mn > Cu > As > Pb > Ni> Cr > Co > Cd and Cu > Cr > Mn > Pb > Ni > Co> Cd > As, respectively. In addition, in Swabi, the order of trace elements was Mn > Cu > Cr > Pb > Ni > As > Co > Cd. Basic statistical reviews of the trace elements in drinking water samples from all areas are presented in Table 1. Although some of the trace elements exhibited much higher levels than others, such as As, Mn, Ni, Cr, and Pb, according to WHO and Pak-EPA standards, the concentrations of Co, Cu, and Cd were within permissible limits. In Lahore, Multan, Vehari, and Jhang, the concentration of As was found to be above the permissible limits set by the WHO [20]. Over the past several decades, elevated arsenic concentrations in groundwater have drawn the attention of many researchers [22]. In South East Asia, this issue is even more pronounced. Several studies revealed that the key arsenic source in groundwater is geological, and that it is mostly derived from the chemical exchange between the groundwater and aquifer sediments [23,24,25]. In this study, all locations are on alluvial terraces located near the river in South and Central Punjab. Few studies have been conducted regarding this contamination source in Pakistan. However, in countries such as Bangladesh and India, which are thought to have similarly reducing geological conditions, more extensive research is available. In Bangladesh, studies have shown that arsenic contamination is more extensive in shallow aquifers. Moreover, the general mechanisms through which arsenic mobilization causes groundwater contamination could be due to arid oxidizing environments with high pH [26,27], reducing environments [28,29], oxidative weathering [30], and geothermal activity [31,32]. It has been suggested that a high concentration of As correlates strongly with soil pH [25], which is consistently elevated throughout the Indus. The mean arsenic level recorded in the present investigation was higher than that found in Pakistan [33] but lower than the As concentrations reported in India [34], China [34], and Bangladesh [35]. Consumption of As-contaminated water on a regular basis has adverse health outcomes including lung cancer, male infertility, skin disorders, and various cardiovascular diseases [36,37]. Mn was found to be within the safe limits for drinking water in most of the areas except in Jhang, where it ranged from 20 to 4917 ng/g. Mn-containing rocks, and mineral weathering and leaching increases its concentration in the aquifers. Furthermore, Mn may also enter groundwater through mineral processing, wastewater discharge, steel emissions, mining activities, fossil fuel combustion, battery manufacturing, fungicides, and fertilizers (MnSO_4_) [38]. The Mn levels reported in the current investigation were higher than they were in studies conducted in Swat [39], Besham [40], Sialkot, and Manchar Lake [41]. The ranges of Ni concentration were beyond the safe limits in Lahore (4–7190 ng/g) and in Vehari (3–195 ng/g). A higher Ni concentration could be due to the ultramafic and mafic industrial activities and the erosion of rocks in the area [42,43]. Ni is a basic constituent of diet, but its elevated concentration results in asthma, lung fibrosis, vomiting, birth defects, skin allergies, conjunctivitis, and respiratory tract cancer [44]. The Ni concentrations recorded were higher than those observed in Nawabshah [45], Peshawar [46], and Faisalabad [47]. Lead also exceeded the safe limits in Lahore, Jhang, and Vehari, with concentrations higher than that recommended by the WHO [20]. Its higher concentration could be due to its extensive usage in agricultural insecticides, mafic and ultramafic rock leaching and weathering, and corrosive and weak plumbing systems [48]. Previous studies have shown a higher Pb level in Swat [15], while a lower level was recorded in Peshawar [46]. The Cr concentrations in water samples were 2593 ± 3250, 5 ± 3.4, 11.6 ± 9.4, 8.03 ± 14.09, 5.79 ± 1.17, and 4.13 ± 2.6 ng/g in selected areas, respectively (Table 1). The highest concentration of Cr was observed in Lahore and was higher than the permissible limits, while all the other areas had a Cr concentration within permissible limits, as found in Sialkot [49], Faisalabad [47], and Peshawar [46]. Cr is used extensively in different industrial activities such as cement dyeing, metal cleaning, leather tanning, and electroplating. Moreover, agricultural activities, and weak and corrosive plumbing may have added Cr into the environment. A high Cr concentration causes several health problems such as tumor formation, a weak immune system, birth defects, and respiratory problems [50].

### 3.2. Chronic Daily Intake and Health Risk Indices of Trace Elements

The chronic daily intake (CDI) values and health risk indices (HRI) of the selected trace elements are shown in Table 2 and Table 3. HRI values were scaled as follows: <1 = no risk; >1 = health risk. HRI values for all elements in all locations were found to be <1, except for those of As in Lahore and Jhang in Central Punjab, and in Vehari and Multan in South Punjab, and also those of Cr and Ni in Lahore. The mean CDIs of Cr in terms of groundwater consumption for adults ranged from 74.08 to 0.118 μg/kg per day from Lahore to Swabi (Table 2). The lowest Cd CDI for adults was recorded at Swabi, while the highest Cd CDI 74.08 μg/kg per day for adults was recorded at Lahore. The health risk indices of Cr showed that they were within safe limits (HRI < 1), but for Lahore, a health risk was found in the region. The mean Mn CDIs in terms of groundwater consumption for adults in the six locations ranged from 0.095 to 19.8 μg/kg per day. The health risk index suggested that all the six locations were within safe limits (HRI < 1), and therefore, no health risk from Mn was found in the selected regions. The highest Co (0.57 μg/kg per day) for adults was recorded in the Lahore region in terms of groundwater consumption. However, the lowest Co CDI was found in Swabi. The HRI showed that all locations were within safe limits in terms of Co concentrations. The lowest Ni CDI (0.026 μg/kg per day) of groundwater for adult consumption was found at Peshawar, while the highest Ni CDI (37.31 μg/kg per day) was recorded in Lahore. Moreover, the HRI values for Ni in the Lahore region were not within safe limits (HRI >1). Likewise, the mean As CDIs ranged from 0.00 to 1.4 μg/kg per day from Lahore to Swabi in terms of groundwater consumption for adults. However, in the current investigation, the HRI values of arsenic showed that most of the areas such as Lahore, Vehari, Multan, and Jhang were not within safe limits (HRI > 1), suggesting high health risks in these regions. Moreover, for Cd and Pb, the HRI values were within the safe limits, suggesting no health risks, as shown in Table 3. CDIs for cadmium ranged from 0.00 to 0.017 μg/kg per day, and CDIs for Pb ranged from 0.03 to 0.42 μg/kg per day. The data in Table 3 demonstrated that the HRI values of As, Cr, and Ni were higher (HRI > 1) in this study as compared to previous studies, suggesting health risks in some of the regions. The HRI results of Cr, Mn, and Ni are similar to those of other studies conducted earlier [39,48].

### 3.3. Trace Elements Pollution Source Analysis

#### 3.3.1. Trace Elements in Different Locations

**Figure 2:**
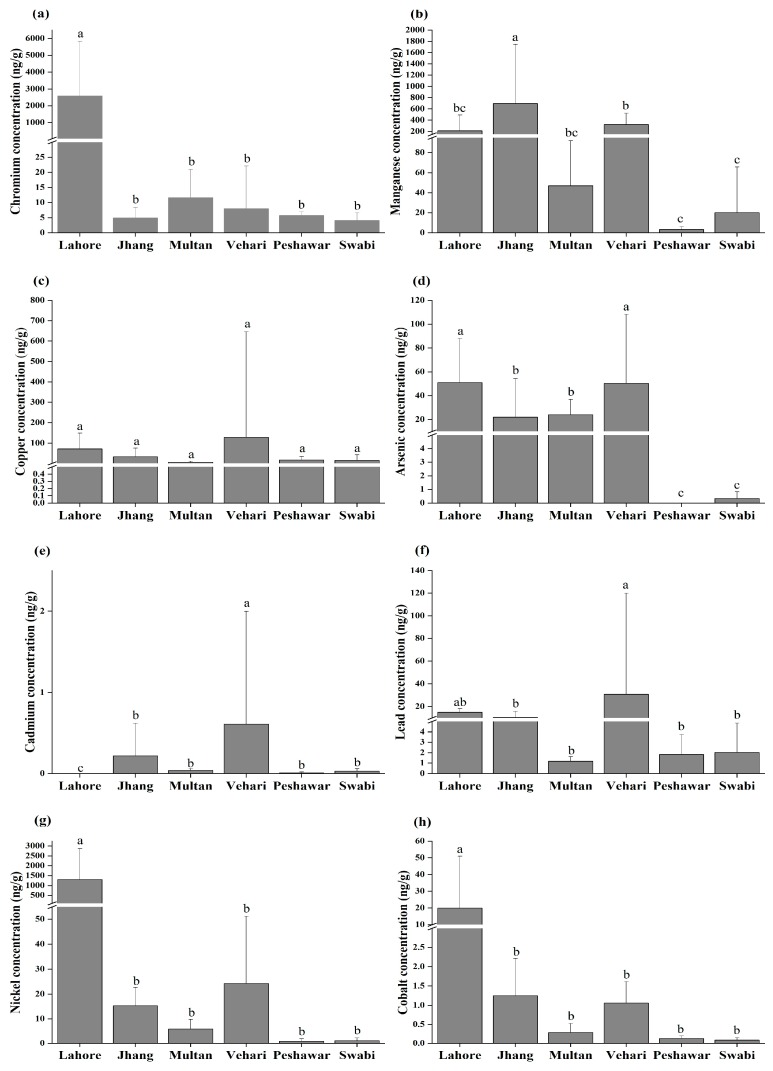
Heavy metals concentrations in different cities of Pakistan, (**a**) Cr, (**b**) Mn, (**c**) Cu, (**d**) As, (**e**) Cd, (**f**) Pb, (**g**) Ni, and (**h**) Co. Error bars represent standard errors of the means (*n* = 20). The least significant differences (LSD_0.05_) are at the 5% level of significance. Different letters on top (**a**–**c**) of each bar show significant differences among different cities.

We performed a statistical analysis on the comparison of selected trace elements in different locations. The analyzed outcomes show significant variation (*p* < 0.05) between different locations, which indicated that different locations contribute differently to the mean trace element concentration in the water (Figure 2). The analysis of variance demonstrated that Cr concentrations were significantly higher (*p* < 0.05) in the drinking water samples collected from the different areas. Statistically remarkable differences were found between Lahore–Jhang, Lahore–Multan, Lahore–Vehari, Lahore–Peshawar, and Lahore–Swabi. However, Jhang–Swabi, Multan–Vehari and Peshawar–Swabi showed non-significant relationships. Correspondingly for Mn, statistically notable differences were found between Jhang–Multan, Jhang–Lahore, Jhang–Peshawar, Jhang–Swabi, Jhang–Vehari, Vehari–Peshawar and Vehari–Swabi. However no significant difference (*p* < 0.05) was found for Cu concentration among different localities. Overall, As concentrations in drinking water samples in Jhang, Lahore, and Vehari showed statistically significant differences (*p* < 0.05). However, Lahore–Vehari, Jhang–Multan, and Peshawar–Swabi showed no significant differences. Likewise, Ni concentration was significantly higher in Lahore as compared to other cities. Pb, Cd, and Co concentrations also showed significant differences.

#### 3.3.2. Pearson Correlation Analysis between Selected Trace Elements

The Spearman correlation test was used to find possible correlations between these trace elements in each zone separately, to discover that either the presence of particular trace elements in a sample facilitates the occurrence of other elements, or if they coexist due to anthropogenic activity in the areas. Table 4 represents the correlation matrix with statistically significant correlations. Although other elements exhibited multiple statistically significant correlations with strong correlation coefficients, no such correlations were found for arsenic in any of the zones. In Central Punjab, the correlation analysis showed positive correlations in some trace element pairs, such as Co–Cr (r = 0.92), Ni–Cr (r = 0.93), Cu–Cr (r = 0.57), Ni–Co (r = 0.96), Cu–Co (r = 0.62), Pb–Co (r = 0.57), and Pb–Ni (r = 0.57). In the case of South Punjab, the correlation analysis revealed positive correlations in several metals, such as Co–Mn (r = 0.63), Cu–Ni (r = 0.66), Cd–Ni (0.57), Pb–Ni (r = 0.73), Pb–Cu(r = 0.87), and Cd–As (r = 0.67). In KPK, strong correlations were observed between different metals, such as Co–Mn (r = 0.55), Ni–Mn (r = 0.59), Ni–Co (r = 0.68), Cd–Co (r = 0.81), Pb–Mn (r = 0.67), Cd–Ni (r = 0.69), Pb–Ni (r = 0.73), and Pb–Cd (r = 0.85). Correlation analyses of all trace elements present in drinking water (except As) from all areas exhibited strong, statistically significant correlations with other elements in all zones. This also shows that the pathway of As into groundwater is not similar to the other elements that correlate with each other, and could be anthropogenic. The absence of correlations among the metals suggests that the contents of these metals are not controlled by a single factor, but rather by a combination of geochemical support phases [51].

#### 3.3.3. Principle Component Analysis

**Figure 3:**
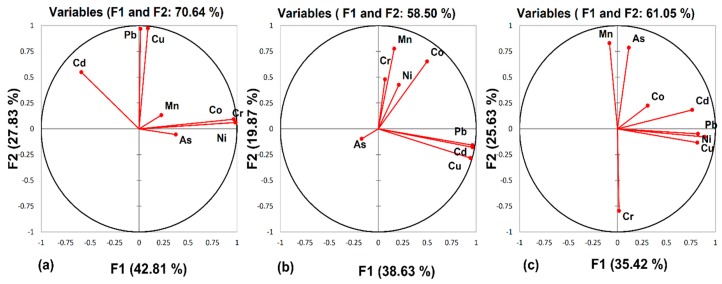
Factor loadings for selected trace elements in the drinking water of (**a**) Central Punjab, (**b**) Southern Punjab, and (**c**) Khyber Pakhtunkhwa, respectively.

Principle component analysis (PCA) was used to compare the compositional and spatial patterns between the examined water samples and the identified latent factors, and to find the possible sources of the trace metals in the water samples. PCA has been widely applied to identify the contribution of anthropogenic and natural sources [52]. The data set was treated by PCA by applying Varimex rotation with Kaiser Normalization as a principal component extraction method. The factor loadings of these different elements with percentages of cumulative variance are given in Figure 3. Two principal components were extracted by PCA in Central Punjab, which was sufficient to cover 70.64% of the total variance from eight elements. In the Central Punjab region, the principal components of Factor 1 accounted (43%) and Factor 2 was recorded (28%) respectively. The elements Cr, Ni, and Co were loaded in Factor 1, while Pb and Cd were loaded in Factor 2. Similarly, As showed a positive correlation in Factor 1 (r = 0.3). Ni and Cr levels may result from industrial activities, as well as mafic and ultramafic rock erosion and weathering in the area. On the other hand, Co sources for the general population mostly originate from industrial activities. Pb is used in many industrial applications, such as solder ammunition, cable sheathing, and battery recycling pigments. Moreover, Pb exposure in water may be due to a weak and corrosive plumbing system. Mn was loaded in both Factor 1 (r = 0.22) and Factor 2 (r = 0.1), but it was not dominant. These results suggest that Factor 1 and Factor 2 have both geological and anthropogenic contributions.

In the case of South Punjab, the total cumulative variance of the two Factors was 58.50%, in which Factor 1 contributed 39% and Factor 2 contributed 20%, with high loadings on Cu (r = 0.9), Cd (r = 0.9), and Pb (r = 0.9) in Factor 1. The Cd level may be due to geological activities, while Pb could be influenced by the plumbing systems in Vehari and Multan. Moreover, Mn (r = 0.7) and Co (r = 0.6) were efficiently loaded in Factor 2. Arsenic showed negative loading in both Factors. The results show that Factor 1 and 2 may indicate both anthropogenic and geologic sources.

When investigating the results of KPK, it was found that the total cumulative variance for the two factors in KPK was 61%. Factor 1 contributed 35% of the total variance, with high loadings of Ni (r = 0.8), Pb (r = 0.8), and Cd (r = 0.7). Moreover, Mn and arsenic were loaded in Factor 2, showing that both may have anthropogenic and geological contributions. The high Ni level could be due to the weathering of rocks.

## 4. Conclusions

After thorough investigation of the drinking water samples collected from groundwater of the investigated areas, the highest concentration was found for Chromium (Cr), followed by Ni > Mn > Cu > As > Pb > Co > Cd. The concentrations of As, Mn, Ni, Cr and Pb were higher than their respective permissible limits set by the Pak-EPA and the WHO, while Cu, Co, and Cd concentrations were within their permissible limits. The HRI and CDI values of As showed that the water is not safe for drinking in Lahore, Vehari, Multan, and Jhang. Similarly, Cr and Ni also exceeded the safe limits in Lahore. However, for Mn, Cu, Co, Cd, and Pb, the HRI values were within the limits. ANOVA analysis indicated that trace element contaminations in different areas varied significantly. The Lahore area has contributed high levels of contamination. It has been observed from the univariate and multivariate statistical analyses that both anthropogenic (Cr, Pb, Ni, Cu, Co, Cd, Mg) and geologic sources (As) were responsible for higher trace element concentrations. To sum off, despite being an essential part of life, As, Cr, and Ni contaminated water may pose a serious health threat to some of the investigated areas. Therefore, it is strongly recommended that water from polluted areas should not be used for drinking purposes without proper treatment. Industrial wastewater disposal in big cities such as Lahore and Multan should be strictly monitored, and all industries should be directed to adapt and follow wastewater treatment measures.

## Figures and Tables

**Table 1:** Basic statistical reviews of the trace element compositions in water samples.

Location	Statistics	Concentration (ng/g) of Selected Trace Elements in Drinking Water Samples (*n* ^a^ = 20)							
		Cr	Mn	Co	Ni	Cu	As	Cd	Pb
**Lahore**	Mean ± SD	2593 ± 3250	212 ± 280	20 ± 31	1306 ± 1566	72 ± 77	51 ± 37	0.00 ± 0.00	15 ± 3.12
	Max–Min	15030–1	130–4	140–5	7190–4	347–0	97–3	0–0	21–9
**Jhang**	Mean ± SD	5 ± 3.4	695 ± 1056	1.25 ± 0.97	15.32 ± 7.34	32.9 ± 42.3	22 ± 32.3	0.22 ± 0.4	10.31 ± 5.6
	Max–Min	17–1	4917–20	5–0	31–2	152–3	135–0	2–0	22–0
**Multan**	Mean ± SD	11.6 ± 9.4	47 ± 45	0.29 ± 0.24	5.98 ± 3.9	6.61 ± 4.9	24 ± 13	0.04 ± 0.022	1.18 ± 0.43
	Max–Min	34–2	281–1	1–0	14–1	20–1	47–0	0.09–0	2–0
**Vehari**	Mean ± SD	8.03 ± 14.09	322.4 ± 203	1.06 ± 0.55	24.22 ± 27	129 ± 517	50.4 ± 58	0.61 ± 1.39	31 ± 89
	Max–Min	66–0	641–3	3–0	195–3	2325–4	224–1	6–0	381–3
**Peshawar**	Mean ± SD	5.79 ± 1.17	3.35 ± 2.6	0.13 ± 0.07	0.94 ± 0.92	17.21 ± 16.8	0.00 ± 0.00	0.01 ± 0.010	1.84 ± 1.9
	Max–Min	8–2	10–1	0–0	3–0	69–2	0–0	0.011–0	7–0
**Swabi**	Mean ± SD	4.13 ± 2.6	20.3 ± 45.5	0.09 ± 0.06	1.10 ± 1.17	16.10 ± 26.4	0.35 ± 0.48	0.03 ± 0.03	2.03 ± 2.8
	Max–Min	11–1	168–1	0–0	4–0	89–3	2–0	0.13–0	12–0
**WHO ^b^**	Permissible limits	50	400	50	70	2000	10	3.00	10
**Pak-EPA ^c^**	Permissible limits	50	500	50	20	2000	50	10	50

^a^ Number of water samples; ^b^ World Health Organization (WHO, 2008); ^c^ Pakistan Environmental Agency (Pak-EPA, 2008).

**Table 2:** Chronic daily intakes (CDI; μg/kg per day) of trace elements through drinking water (*n* = 120).

Location	Cr	Mn	Co	Ni	Cu	As	Cd	Pb
Lahore	74.08	6.057	0.571	37.31	2.05	1.4	0.00	0.42
Jhang	0.142	19.8	0.035	0.43	0.94	0.6	0.006	0.29
Multan	0.3314	1.342	0.008	0.170	0.188	0.68	0.001	0.0337
Vehari	0.229	9.2	0.030	0.692	3.68	1.44	0.017	0.885
Peshawar	0.165	0.095	0.0037	0.026	0.49	0.00	0.0002	0.052
Swabi	0.118	0.58	0.0025	0.031	0.46	0.01	0.00085	0.058

**Table 3:** Health risk indices (HRI) for different elements in the studied areas through drinking water (*n* = 120).

Location	Cr	Mn	Co	Ni	Cu	As	Cd	Pb
Lahore	25 *	4.3 × 10^−2^	0.4	1.8 *	5.1 × 10^−2^	4.8 *	0.00	0.12
Jhang	4.7 × 10^−2^	1.4 × 10^−1^	2.5 × 10^−2^	2.1 × 10^−2^	2.3 × 10^−2^	2.0 *	6.2 × 10^−3^	8.4 × 10^−2^
Multan	0.11	9.5 × 10^−3^	5.9 × 10^−3^	8.5 × 10^−3^	4.7 × 10^−3^	2.28 *	1.1 × 10^−3^	9.6 × 10^−3^
Vehari	7.0 × 10^−2^	6.5 × 10^−2^	2.1 × 10^−2^	3.4 × 10^−2^	9.2 × 10^−2^	4.8 *	1.7 × 10^−2^	0.25
Peshawar	5.5 × 10^−3^	6.8 × 10^−3^	2.6 × 10^−3^	1.3 × 10^−3^	1.2 × 10^−2^	0.00	2.8 × 10^−4^	1.5 × 10^−2^
Swabi	3.9 × 10^−2^	4.1 × 10^−3^	1.8 × 10^−3^	1.5 × 10^−3^	1.1 × 10^−2^	3.3 × 10^−2^	8.5 × 10^−4^	1.6 × 10^−2^

* Represents the high HRI values showing health risk.

**Table 4:** Correlation matrixes of selected trace elements in the water samples from Central Punjab, Southern Punjab and Khyber Pakhtunkhwa (*n* = 120).

		Cr	Mn	Co	Ni	Cu	As	Cd	Pb
**Central** **Punjab**	**Cr**	1.000							
	**Mn**	0.285	1.000						
	**Co**	0.922 **	0.375 *	1.000					
	**Ni**	0.935 **	0.375 *	0.965 **	1.000				
	**Cu**	0.572 **	0.149	0.622 **	0.656 **	1.000			
	**As**	0.180	0.047	0.070	0.145	0.000	1.000		
	**Cd**	−0.269	0.465 **	−0.151	−0.168	−0.139	−0.329 *	1.000	
	**Pb**	0.495 **	0.187	0.573 **	0.578 **	0.774 **	0.159	−0.116	1.000
**South** **Punjab**	**Cr**	1.000							
	**Mn**	−0.155	1.000						
	**Co**	0.178	0.631 **	1.000					
	**Ni**	0.111	0.378 *	0.449 **	1.000				
	**Cu**	0.226	0.293	0.419 **	0.668 **	1.000			
	**As**	−0.377 *	0.279	0.060	0.266	0.098	1.000		
	**Cd**	−0.230	0.134	−0.015	0.572 **	0.280	0.675 **	1.000	
	**Pb**	0.117	0.363 *	0.483 **	0.731 **	0.877 **	0.211	0.446 **	1.000
**Khyber** **Pakhtunkhwa**	**Cr**	1.000							
	**Mn**	0.032	1.000						
	**Co**	−0.032	0.552 **	1.000					
	**Ni**	0.321 *	0.592 **	0.680 **	1.000				
	**Cu**	0.384 *	0.336 *	0.376 *	0.577 **	1.000			
	**As**	−0.227	0.125	0.011	0.182	−0.125	1.000		
	**Cd**	−0.073	0.468 **	0.811 **	0.696 **	0.446 **	0.116	1.000	
	**Pb**	−0.112	0.671 **	0.830 **	0.730 **	0.403 **	0.147	0.850 **	1.000

* Correlation is significant at the 0.05 level (2-tailed); ** Correlation is significant at the 0.01 Level (2-tailed).
